# Sex influences eQTL effects of SLE and Sjögren’s syndrome-associated genetic polymorphisms

**DOI:** 10.1186/s13293-017-0153-7

**Published:** 2017-10-25

**Authors:** Magdalena Lindén, Jorge I. Ramírez Sepúlveda, Tojo James, Gudny Ella Thorlacius, Susanna Brauner, David Gómez-Cabrero, Tomas Olsson, Ingrid Kockum, Marie Wahren-Herlenius

**Affiliations:** 1 Unit of Experimental Rheumatology, Department of Medicine, Karolinska University Hospital, Karolinska Institutet, Stockholm, Sweden; 2 Unit of Neuroimmunology, Department of Clinical Neuroscience, Karolinska University Hospital, Karolinska Institutet, Stockholm, Sweden; 3 Unit of Computational Medicine, Karolinska University Hospital, Karolinska Institutet, Stockholm, Sweden; 4 Unit of Clinical Epidemiology, Department of Medicine, Karolinska University Hospital, Karolinska Institutet, Stockholm, Sweden; 5 Science for Life Laboratory, 17121 Solna, Sweden; 6 Mucosal and Salivary Biology Division, King’s College London Dental Institute, London, SE1 9RT UK

**Keywords:** Sjögren’s syndrome, SLE, Autoimmunity, Sex difference, eQTL

## Abstract

**Background:**

Systemic lupus erythematosus (SLE) and primary Sjögren’s syndrome (pSS) are autoimmune disorders characterized by autoantibodies, dysregulated B cells, and notably high female-to-male incidence ratios. Genome-wide association studies have identified several susceptibility SNPs for both diseases. Many SNPs in the genome are expression quantitative trait loci (eQTLs), with context-dependent effects. Assuming that sex is a biological context, we investigated whether SLE/pSS SNPs act as eQTLs in B cells and used a disease-targeted approach to understand if they display sex-specific effects.

**Methods:**

We used genome-wide genotype and gene expression data from primary B cells from 125 males and 162 females. The MatrixEQTL R package was used to identify eQTLs within a genomic window of 2 Mb centered on each of 22 established SLE and/or pSS susceptibility SNPs. To find sex-specific eQTLs, we used a linear model with a SNP * sex interaction term.

**Results:**

We found ten SNPs affecting the expression of 16 different genes (FDR < 0.05). rs7574865-*INPP1*, rs7574865-*MYO1B*, rs4938573-*CD3D*, rs11755393-*SNRPC*, and rs4963128-*PHRF1* were novel observations for the immune compartment and B cells. By analyzing the SNP * sex interaction terms, we identified six genes with differentially regulated expression in females compared to males, depending on the genotype of SLE/pSS-associated SNPs: *SLC39A8* (*BANK1* locus), *CD74* (*TNIP1* locus), *PXK*, *CTSB* (*BLK/FAM167A* locus), *ARCN1* (*CXCR5* locus), and *DHX9* (*NCF2* locus).

**Conclusions:**

We identified several unknown sex-specific eQTL effects of SLE/pSS-associated genetic polymorphisms and provide novel insight into how gene-sex interactions may contribute to the sex bias in systemic autoimmune diseases.

**Electronic supplementary material:**

The online version of this article (10.1186/s13293-017-0153-7) contains supplementary material, which is available to authorized users.

## Background

Autoimmune disorders such as SLE and pSS have a multifactorial etiology. Multiple environmental as well as intrinsic risk factors interplay with each other and an individual’s genetic makeup in different combinations over time in order to cause disease. Smoking, infection, and textile dust have been identified as extrinsic factors interacting with the host genome to increase the risk of autoimmune disease [1–3], while adolescent obesity is the only intrinsic factor to date with a proven genetic interaction to influence autoimmune disease risk [4].

The majority of autoimmune diseases, and especially the rheumatic diseases, are more common in women than in men [5]. Systemic lupus erythematosus (SLE) and primary Sjögren’s syndrome (pSS) are clinically and molecularly related systemic autoimmune disorders characterized by autoantibodies and dysregulated B cells. Both diseases have among the highest observed female-to-male ratios compared to other autoimmune conditions (approximately 9:1 for SLE [6] and 9:1 ranging to 20:1 for pSS [7, 8]). The roles of sex hormones and the sex chromosomes have been extensively investigated [9–11], but the strong sex bias remains poorly understood.

Although the etiology of SLE and pSS is unknown, genetics play a significant role. Through genome-wide association studies (GWAS), a growing number of single nucleotide polymorphisms (SNPs) that confer disease susceptibility are being identified for both diseases [12–18]. There is a certain overlap of genetic risk variants between different autoimmune disorders, also between rheumatic diseases, and many of the loci implicated in SLE have also been identified in pSS [17, 18]. Most of the disease-associated SNPs of complex conditions are located in non-coding sequences and are thought to affect regulatory genetic elements, leading to altered transcriptional activity, splicing, and epigenetic marks [19, 20]. Expression quantitative trait loci (eQTLs) are widespread in the genome [21] and likely to account for a substantial part of the causal genetic effects contributing to disease [22, 23]. In fact, several publications show that the expression of a number of genes is affected by SNPs in SLE- and pSS-associated regions. It is becoming increasingly clear that a large proportion of eQTLs present context-dependent effects relating to biological factors such as cell type and activation state [24–26].

Investigating the functional outcome of genetic associations linked to disease will give novel insight to pathogenesis. As there is no difference of the frequency of these pSS and SLE disease-associated SNPs between women and men in the general population, but a much higher likelihood for the diseases to develop in women carrying the SNPs, we hypothesized that the context female sex would influence the functional impact of the genetic polymorphism. Previous investigations of sexually dimorphic eQTLs have been performed in heterogeneous, whole tissue samples [27, 28], and given the central role of B cells in both pSS and SLE pathogenesis, we performed our analysis in an expression dataset derived from purified B cells. We first investigated whether SLE/pSS disease-associated SNPs act as eQTLs in B cells. Further, to better understand the difference between sexes in systemic autoimmune diseases and assuming that sex is a biological context, we thereafter applied a statistical model that takes the interaction between SNPs and sex into account and examined whether eQTL effects differ between women and men in the disease-associated loci.

## Methods

### Study subjects, gene expression array data, and SNP genotypes

Microarray-based CD19^+^ B cell mRNA expression and genotype data from 287 healthy volunteers (age range 18–62 years; median age 33.1 years) was obtained from a study published by Fairfax et al. [24]. Sampling and laboratory procedures are described in detail elsewhere [24]. In short, the Illumina HumanHT-12 v4 BeadChip gene expression array platform was used for total RNA quantification and the expression data was obtained as log-2 transformed values. The Illumina Human OmniExpress-12v1.0 BeadChips, NCBI36 Build, were used for genomic DNA genotyping, and upon granted access, genotype data was downloaded from the European Genome-Phenome Archive as AGCT-coded SNP data.

### SLE- and pSS-associated SNPs

The SNPs were chosen from the current literature of genetic association studies in SLE and/or pSS, with the criteria of reaching genome-wide significance (*p* < 5.0 × 10^−8^) and being the first reported and/or top-associated SNP within the associated region, and/or being genotyped in the Fairfax et al.’s study [24]. SNPs in the *HLA* region were excluded due to low allele frequencies in the dataset (≥ 3 individuals for each associated *HLA* allele were required per genotype group). For SNPs that were not present on the Illumina genotyping chip used by Fairfax et al., proxy SNPs with high linkage disequilibrium (LD) (*r* ^2^ > 0.8) were identified using the SNAP Proxy search tool (SNP dataset: 1000 Genomes Pilot 1; Population panel: Caucasian (CEU); *r* ^2^ threshold 0.8; distance limit 500) [29]. Proxy SNPs and *r* ^2^ values in relation to the corresponding SLE/pSS-associated SNP are found in Table 1. *r* ^2^ values for SLE- and pSS-associated SNPs in the same region were retrieved using HaploReg v.4.1 (1000 Genomes Phase 1, CEU population) [30].

**Table 1:** List of pSS- and/or SLE-associated SNPs used in the eQTL analyses

Chr	SNP	Proxy SNP for top associated SNP (*r* ^2^)	Gene region	MAF^b^males/females	Disease association	Disease risk allele^a^	OR	Assoc P	Reference
1	rs3024505		*IL-10*	0.17/0.14	SLE	A	1.19	4.00 × 10^−8^	Gateva et al. [14]
1	rs10911363	rs12753665 (0.821)	*NCF2*	0.26/0.29	SLE	T(A)	1.23	2.87 × 10^−10^	Cunninghame Graham et al. [16]
2	rs1990760		*IFIH1*	0.42/0.42	SLE	T	1.17	1.63 × 10^−8^	Cunninghame Graham et al. [16]
2	rs7574865		*STAT4*	0.27/0.20	SLE	T	1.57	1.40 × 10^−10^	Gateva et al. [14]
3	rs485497		*IL12A*	0.49/0.51	pSS	A	1.3	1.17 × 10^−10^	Lessard et al. [17]
3	rs6445975		*PXK*	0.23/0.26	SLE	C	1.25	7.10 × 10^−9^	Harley et al. [13]
4	rs10516487	rs4637409 (1)	*BANK1*	0.35/0.30	SLE	G(A)	1.38	3.74 × 10^−10^	Kozyrev et al. [15]
5	rs7708392	rs10036748 (1)	*TNIP1*	0.26/0.25	SLE	C(T)	1.27	3.80 × 10^−13^	Gateva et al. [14]
6	rs6568431		*PRDM1/ATG5*	0.38/0.40	SLE	A	1.2	7.10 × 10^−10^	Gateva et al. [14]
6	rs5029939		*TNFAIP3*	0.04/0.05	SLE	G	2.29	2.86 × 10^−12^	Graham et al. [58]
6	rs11755393		*UHRF1BP1*	0.32/0.34	SLE	G	1.17	2.20 × 10^−8^	Gateva et al. [14]
7	rs2070197	rs10488631 (0.938)	*IRF5/TNPO3*	0.10/0.10	SLE	C(C)	1.88	5.80 × 10^−24^	Gateva et al. [14]
7	rs4728142		*IRF5/TNPO3*	0.45/0.46	pSS	C(A)	1.31	2.73 × 10^−19^	Lessard et al. [17]
7	rs849142		*JAZF1*	0.49/0.50	SLE	T	1.19	1.50 × 10^−9^	Gateva et al. [14]
8	rs13277113		*BLK/C8orf13*	0.25/0.25	SLE	A	1.39	1.00 × 10^−10^	Hom et al. [12]
8	rs2736345	rs922483 (1)	*BLK/C8orf13*	0.29/0.29	pSS	G(T)	1.16	4.97 × 10^−10^	Lessard et al. [17]
8	rs7829816		*LYN*	0.19/0.21	SLE	T	1.23	5.40 × 10^−9^	Harley et al. [13]
11	rs2732552		*CD44, PDHX*	0.40/0.49	SLE	C	1.17	1.82 × 10^−9^	Lessard et al. [59]
11	rs7119038	rs4938573 (0.865)	*CXCR5*	0.22/0.22	pSS	A(T)	1.21	1.10 × 10^−8^	Lessard et al. [17]
11	rs4963128		*IRF7/PHRF1/KIAA1542*	0.36/0.29	SLE	C	1.22	3.00 × 10^−10^	Harley et al. [13]
16	rs11644034		*IRF8*	0.22/0.23	SLE	G	1.22	2.72 × 10^−9^	Lessard et al. [60]
16	rs9888739		*ITGAM-ITGAX*	0.09/0.12	SLE	T	1.62	1.61 × 10^−23^	Harley et al. [13]

*Chr* chromosome, *SNP* single nucleotide polymorphism, *OR* odds ratio, *Assoc P p* value for the disease association

^a^Allele in parenthesis denotes the corresponding allele for the proxy SNP

^b^Minor allele frequency (MAF) in this study dataset

### Imputation of sex of the genotyped individuals and confirmation by expression analysis

We defined the sex of each individual using the “- -impute-sex” command in PLINK v. 1.07. This tool imputes the sex codes based on SNP data by estimating the heterozygosity rates of the X chromosome [31]. To further confirm the sex of the samples, we utilized the MicroArray Sample Sex Identifier R package (massiR) which predicts the sex of a given sample based on the variance of expression levels of Y chromosome genes probes. The probes with higher expression variance across the samples will be indicative of sex variation and, thus, can be used for sex classification.

When we defined the sex of the samples with PLINK or massiR, the same results with a perfect match was obtained.

### Population substructure and principal component analysis

Genotypes for 3736 ancestry informative SNPs were used for principal component analysis (PCA) by running the smartpca program from Eigensoft using standard settings, Additional file 1: Figure S1. Five population outliers were identified. Due to this low number, none of them was removed from the analysis. None of the PCs was significant. There was no need to correct the analysis for PCA vectors, since the population was so homogenous.

### Filtering of expression data

Genomic positions of the Illumina probes were retrieved from NCBI Reference Sequence build 36 utilizing the Illumina HumanHT-12 v4 BeadChip probe information file obtained from Illumina. We obtained SNP coordinates in NCBI build 36 by using the UCSC liftOver tool, http://genome.ucsc.edu/cgi-bin/hgLiftOver (from NCBI build 38 coordinates given in dbSNP) and applying the default parameters. Genes within the range of ± 1 Mb of the SNP of interest were identified using BioMart (http://www.biomart.org/), and probe IDs of the corresponding genes were retrieved from the expression datasets. Among the Y chromosome non-recombining region genes with the highest male-to-female expression ratio in the B cell expression dataset, *EIF1AY* had the highest mean log-2 value in females. We therefore used it to set the threshold for gene expression in the whole dataset. Probes with ≥ 20% of samples below the expression cutoff were excluded from further analysis. We normalized the log2 expression values by transformation to *z* scores.

### General *cis* eQTL analysis

First, using R, we investigated the regression assumptions of linearity, homoscedasticity, and normality of the transformed and scaled expression data for all genes 1 Mb up- and downstream of each included SNP, prior to applying the linear model in subsequent eQTL analyses. This genomic distance is commonly used for studies of *cis-*eQTL effects.

For analysis of eQTL effects in the full cohort, we used the standard method applied in the MatrixEQTL R package [32]. We used sex as a covariate and did not take *trans* eQTL associations into account. For significance, we used a false discovery rate (FDR) of < 0.05 as a cutoff. The number of probes and genes analyzed for each SNP are given in Additional file 2: Table S1.

### Analysis of differential *cis* eQTL effects in males and females

An interaction term to estimate the joint effect of SNP genotype and sex was added to the regression model and was implemented using the “lm” function in R as follows:

lm(Gene Expression ~ SNP genotype + Sex + SNP genotype*Sex).

The “SNP genotype” factor in the argument of the lm model formula was coded based on the additive genetic effect with values 0, 1, 2, for the number of risk alleles, and “Sex” with values 0, 1, for male or female. Normalized *z* score values were used for “Gene Expression.”

In order to exclude potential outlier-driven results and to correct for multiple comparisons, we permuted each SNP genotype 10,000 times and calculated a permutation-based FDR for the SNP genotype*Sex interaction term coefficient according to the SAMseq method [33], which in turn is based on the FDR calculation described by Storey and Tibshirani [34]. The permuted *p* value and FDR cutoff was set to < 0.05.

### Comparisons with published eQTLs

HaploReg v.4.1 (1000 Genomes Phase 1, CEU population) [30] was searched for published eQTL results in any tissue to compare with eQTLs found in this study. Any SNP with *r* ^2^ > 0.8 and displaying an eQTL effect was considered as a published result.

### LD SNP interaction analysis

SNPs in LD with the sex-biased eQTLs were identified using the SNAP Proxy search tool (SNP dataset: 1000 Genomes Pilot 1; Population panel: Caucasian (CEU); *r* ^2^ threshold 0.8; distance limit 500) [29] and extracted from the Fairfax et al. genotype data. LD SNPs and *r* ^2^ values in relation to the corresponding SLE-associated SNP are found in Additional file 3: Table S2. For the rs12753665-DHX9 association only, a SNP with *r* ^2^ = 0.6 was available. rs13277113 and rs922483 are in themselves in high LD with each other, and only one additional proxy SNP could be found for both of them. Therefore, six proxy SNPs were included in the analysis.

### Type 1 diabetes-related polymorphisms

Analysis of differential *cis* eQTL effects in males and females of polymorphisms associated with type 1 diabetes (T1D) was performed as described for SLE/pSS SNPs described above. SNPs were chosen from the current literature of genetic association studies in T1D [35], with the criteria of reaching genome-wide significance (*p* < 5.0 × 10^−8^) and being the first reported and/or top-associated SNP within the region. We also requested the SNP was not associated with any other disease with higher female:male ratio, leaving 15 SNPs for analysis (rs41176, rs229527, rs313841, rs941576, rs1048956, rs1456988, rs2194225, rs4422634, rs6043409, rs6427859, rs7202877, rs7576541, rs9585056, rs10116772, rs11980379).

## Results

### SLE- and pSS-associated eQTLs in B cells

We performed a *cis* eQTL analysis in B cells from a total of 287 healthy volunteers in 2 Mb genomic regions centered on 22 non-*HLA* SNPs with reported genome-wide association with SLE or pSS (Table 1). Ten SNPs displayed significant eQTL effects (FDR < 0.05), affecting a total of 16 different genes (Table 2). rs13277113 and rs922483 are in high LD (*r* ^2^ = 0.83) and both displayed eQTL effects of similar magnitude for *FAM167A* and *BLK.* For *FAM167A*, *SMG7*, *IRF5*, *SNRPC*, *TMEM80*, and *FDFT1*, different Illumina probes mapping to the same gene transcripts generated similar results. Our results agree with the direction of the eQTL effect in relation to the risk alleles reported in other studies [17, 24, 36, 37], with the exception of *IRF5* in relation to the rs4728142 risk allele as reported in the GTEx database.

**Table 2:** SLE- and/or pSS-associated eQTLs in B cells

Chr	SNP	Reported gene(s) associated with SLE/pSS	eQTL gene^a^	Expression vs risk allele	P	FDR	Reference
8	rs13277113	*BLK/FAM167A*	*FAM167A*	Increased	2.13 × 10^−71^	4.12 × 10^−69^	Hom et al. [12], Lessard et al. [17], Fairfax et al. [24], GTEx v6 [36] (immune)
8	rs922483	*BLK/FAM167A*	*FAM167A*	Increased	5.17 × 10^−71^	5.00 × 10^−69^	Hom et al. [12], Lessard et al. [17], Fairfax et al. [24], GTEx v6 [36] (immune)
7	rs4728142	*IRF5/TNPO3*	*IRF5*	Decreased	2.69 × 10^−20^	1.04 × 10^−18^	Fairfax et al. [24], GTEx v6 [36] (immune)
1	rs12753665	*NCF2*	*SMG7*	Decreased	2.69 × 10^−19^	8.69 × 10^−18^	Fairfax et al. [24], GTEx v6 [36] (immune)
8	rs13277113	*BLK/FAM167A*	*BLK*	Decreased	7.19 × 10^−15^	1.99 × 10^−13^	Hom et al. [12], Lessard et al. [17], Fairfax et al. [24], GTEx v6 [36] (immune)
8	rs922483	*BLK/FAM167A*	*BLK*	Decreased	1.04 × 10^−12^	2.51 × 10^−11^	Hom et al. [12], Lessard et al. [17], Fairfax et al. [24], GTEx v6 [36] (immune)
11	rs4963128	*IRF7/PHRF1/KIAA1542*	*IRF7*	Increased	1.97 × 10^−7^	4.24 × 10^−6^	Westra et al. [37]
2	rs7574865	*STAT4*	*INPP1*	Increased	6.05 × 10^−5^	1.17 × 10^−3^	Not reported^b^
6	rs11755393	*UHRF1BP1*	*SNRPC*	Decreased	8.33 × 10^−5^	1.54 × 10^−3^	GTEx v6 [36] (not immune)
7	rs4728142	*IRF5/TNPO3*	*TNPO3*	Decreased	2.18 × 10^−4^	3.83 × 10^−3^	Westra et al. [37]
11	rs4963128	*IRF7/PHRF1/KIAA1542*	*TMEM80* (probe picks up 4 transcripts)	Decreased	2.37 × 10^−4^	3.99 × 10^−3^	Lappalainen et al. [21], Westra et al. [37], GTEx v6 [36] (immune)
2	rs7574865	*STAT4*	*MYO1B*	Decreased	8.83 × 10^−4^	0.014	Not reported^b^
11	rs4963128	*IRF7/PHRF1/KIAA1542*	*TMEM80* (probe picks up 2 transcripts)	Decreased	1.45 × 10^−3^	0.023	Lappalainen et al. [21], Westra et al. [37], GTEx v6 [36] (immune)
7	rs10488631	*IRF5/TNPO3*	*IRF5*	Decreased	2.00 × 10^−3^	0.028	Lappalainen et al. [21]
11	rs4938573	*CXCR5*	*CD3D*	Increased	2.12 × 10^−3^	0.028	Not reported^b^
8	rs922483	*BLK/FAM167A*	*FDFT1* (probe picks up 14 transcripts)	Increased	2.12 × 10^−3^	0.028	Zeller et al. [61]
6	rs11755393	*UHRF1BP1*	*UHRF1BP1*	Increased	2.26 × 10^−3^	0.028	GTEx v6 [36] (immune)
11	rs4963128	*IRF7/PHRF1/KIAA1542*	*PHRF1*	Decreased	3.00 × 10^−3^	0.036	GTEx v6 [36] (not immune)
8	rs13277113	*BLK/FAM167A*	*FDFT1* (probe picks up 11 transcripts)	Increased	3.31 × 10^−3^	0.038	Zeller et al. [61]
11	rs4963128	*UHRF1BP1*	*LRRC56*	Increased	3.60 × 10^−3^	0.040	Westra et al. [37]
3	rs6445975	*PXK*	*DNASE1L3*	Decreased	4.53 × 10^−3^	0.049	Fairfax et al. [24]

*Chr* chromosome, *SNP* single nucleotide polymorphism, *eQTL* expression quantitative locus, *P p* value, *FDR* false discovery rate

^a^For significant eQTLs where different probes detected the same transcript, only the result for the most significant probe is included in the table

^b^For rs7574865 no other genes in the region have been reported; for rs4938573 a cis-eQTL effect was reported for *TREH* in liver and for *AP002954.6* in the tibial nerve and the thyroid gland (GTeX, [36])

We found three eQTL associations which, to our knowledge, have not been published before for any cell type nor has any SNP with *r* ^2^ > 0.8 been reported for the same eQTL effect: rs7574865-*INPP1*, rs7574865-*MYO1B*, and rs4938573-*CD3D.* rs11755393-*SNRPC* and rs4963128-*PHRF1* were only reported in the GTEx database [36], however, not in any immune related tissue and are thus novel observations for the immune compartment and B cells (Table 2, Additional file 4: Figure S2)*.* For rs4938573, an eQTL effect on two genes in the same region, *TREH* and *AP002954*, has been reported in liver tissue [36] and tibial nerve as well as thyroid gland, respectively [36].

In summary, we identified five novel eQTL associated genes in B cells for SLE- and pSS-related genetic polymorphisms and confirmed 11 previously reported eQTL associations.

### Differential SLE- and pSS-associated eQTL effects in males and females

With the aim of testing whether there are eQTL effects of SLE- and pSS-associated SNPs that differ significantly between males and females (i.e., either an eQTL effect that is stronger or exists only in one of the sexes, or eQTL effects in both sexes, but with differing directions), we applied a linear model including the SNP genotype and the sex of the subject (125 males and 162 females in our study) as separate terms, as well as an interaction term for the joint effect of SNP genotype and sex. By analyzing the significance of the “SNP genotype * Sex” interaction coefficient, we identified seven SLE- and/or pSS-associated SNPs with eQTL effects on six different genes which differed significantly between males and females (FDR < 0.05) and with substantial effect sizes reflected by *β* values up to 0.66 (range − 1 to 1) (Table 3).

**Table 3:** SLE- and/or pSS-associated SNPs displaying differential eQTL effects in females compared to males

Chr	SNP	Reported gene(s) associated with SLE/pSS	eQTL Gene	Probe ID	Intercept	P SNP	P Sex	P SNP × Sex	β value SNP × Sex	FDR SNP × Sex
4	rs4637409	*BANK1*	*SLC39A8*	ILMN_1695316	8.13 × 10^−3^	0.016	1.20 × 10^−4^	2.10 × 10^−4^	0.66	7.90 × 10^−3^
5	rs10036748	*TNIP1*	*CD74*	ILMN_2379644	0	0.025	0.638	2.40 × 10^−3^	− 0.55	0.019
3	rs6445975	*PXK*	*PXK*	ILMN_1815063	1.34 × 10–^261^	0.261	0.020	6.00 × 10^−3^	− 0.54	0.022
8	rs922483	*BLK/FAM167A*	*CTSB*	ILMN_1696360	2.85 × 10^−305^	0.104	0.257	6.90 × 10^−3^	0.51	0.028
8	rs13277113	*BLK/FAM167A*	*CTSB*	ILMN_1696360	1.26 × 10^−232^	0.068	5.00 × 10^−3^	8.60 × 10^−3^	0.50	0.029
11	rs4938573	*CXCR5*	*ARCN1*	ILMN_1699703	0.065	0.099	0.014	0.029	0.46	0.042
1	rs12753665	*NCF2*	*DHX9*	ILMN_1690965	0.442	0.126	0.158	0.013	− 0.45	0.044

*Chr* chromosome, *SNP* single nucleotide polymorphism, *eQTL* expression quantitative locus, *P p* value, *β value* regression coefficient, *FDR* false discovery rate

**Fig. 1:**
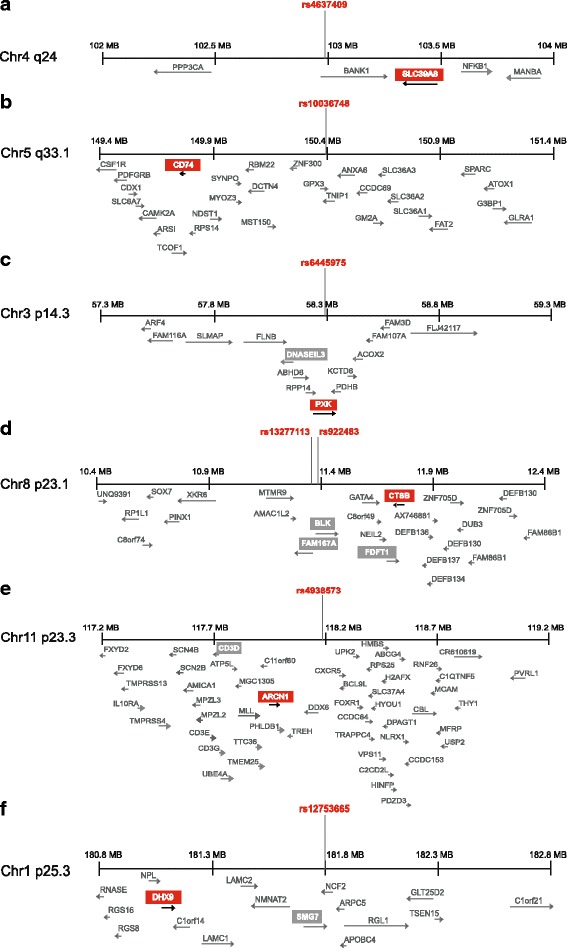
Chromosome and DNA strand location of SNPs with significant sex-influenced eQTL effect and surrounding genes in a ± 1 Mb interval. Genes with significantly different sex-influenced expression are labeled in red, and genes with non-sex influenced expression are labeled gray. **a** rs4637409, proxy SNP (*r* ^2^ = 1) for the SLE-associated rs10516487 (*BANK1* locus) with a sex-dependent eQTL effect on *SLC39A8* expression*,* located on chromosome 4*,* **b** rs10036748, proxy SNP (*r* ^2^ = 1) for the SLE-associated rs7708392 (*TNIP1* locus) with a sex-dependent eQTL effect on *CD74* expression*,* located on chromosome 5. **c** The SLE-associated rs6445975 (*PXK* locus), with a sex-dependent eQTL effect on *PXK* expression, located on chromosome 3. **d** rs922483 (proxy SNP for the pSS-associated rs2736345, *r* ^2^ = 1) and the SLE-associated rs13277113 (*BLK/FAM167A* locus) with sex-dependent eQTL effects on *CTSB* expression*.* **e** rs4938573, proxy SNP (*r* ^2^ = 0.865) for the pSS-associated rs7119038 (*CXCR5* locus) with a sex-dependent eQTL effect on *ARCN1* expression. **f** rs12753665, proxy SNP (*r* ^2^ = 0.821) for the SLE-associated rs10911363 (*NCF2* locus) with a sex-dependent eQTL effect on *DHX9* expression

**Fig. 2:**
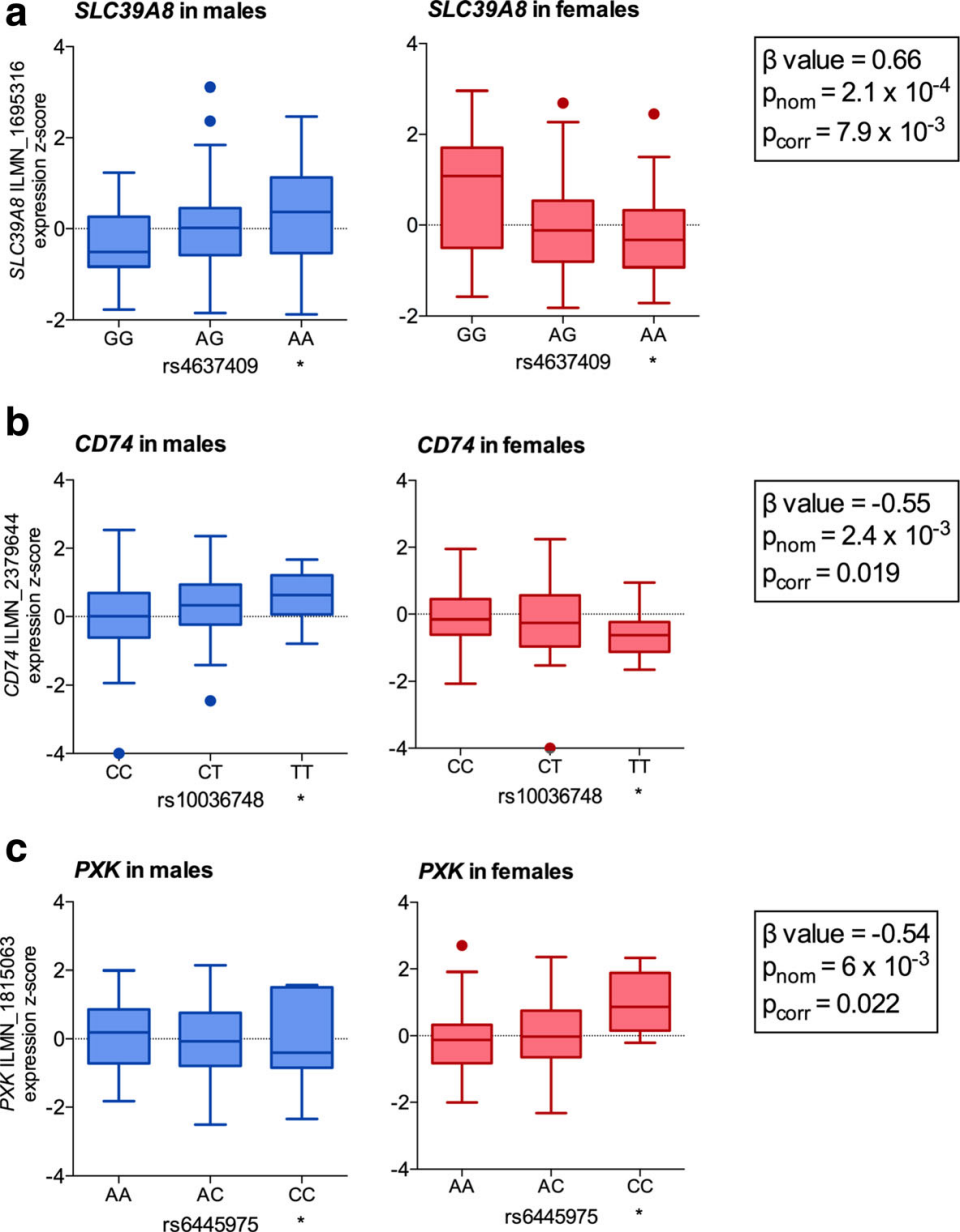
eQTL effects of disease polymorphisms differ between males (left column, blue boxes) and females (right column, red boxes). **a** Genotypic effects of the proxy SNP rs4637409 (*r* ^2^ = 1) for the SLE-associated rs10516487 (*BANK1* locus) on expression of *SLC39A8* (ILMN_1695316 probe) in males (*p* = 0.041) and females (*p* = 0.052). **b** Genotypic effects of the proxy SNP rs10036748 (*r* ^2^ = 1) for the SLE-associated rs7708392 (*TNIP1* locus) on expression of *CD74* (ILMN_2379644 probe) in males (*p* = 0.065) and females (*p* = 0.048)*.* **c** Genotypic effects of the SLE-associated rs6445975 (*PXK* locus) on expression of *PXK* (ILMN_1815063 probe) in males (*p* = 0.60) and females (*p* = 0.0029)*.* The rightmost genotype group in each graph denotes the homozygous group of the disease risk allele and is marked by an asterisk. Statistics in boxes represent the SNP * Sex interaction term analysis

The joint effect of “SNP genotype * Sex” contributed more significantly to the linear model than the single effect of “SNP genotype” for the expression of all these six genes. For rs4637409-*SLC39A8*, rs13277113-*CTSB*, and rs4938573-*ARCN1*, the single effect of “Sex” to the linear model was comparable to the effect of “SNP genotype * Sex,” while the contribution of the interaction term was markedly more significant for rs10036748-*CD74*, rs6445975-*PXK*, rs922483-*CTSB*, and rs12753665-*DHX9* than any of the single coefficient contributions. Figure 1 shows the genomic maps for the significantly differing eQTL effects in females compared to males. rs4637409 is located in an intron of *BANK1*; however, the differential eQTL effect in males and females was observed for *SLC39A8*, the first gene located approximately 400 kb 3′ of *BANK1* (Fig. 1a). *CD74* is located almost 1 Mb away from rs10036748 (Fig. 1b). *PXK* is differentially regulated in a sex-dependent manner by rs6445975, located in an intron of the same gene (Fig. 1c), for which a sex-independent eQTL effect of rs6445975 has been published previously [21, 36]. *CTSB*, *ARCN1*, and *DHX9* are located 250 kb–1 Mb from the disease risk SNP (Fig. 1d–f). In males, the SLE risk alleles of rs4637409 and rs10036748 were associated with higher expression of *SLC39A8* and *CD74*, respectively, while the direction of this eQTL effect was the opposite in females, where the SLE risk alleles were associated with lower gene expression (Fig. 2a, b). rs6445975 showed no eQTL effect in males, while in females the SLE risk allele was associated with higher expression of *PXK* (Fig. 2c). The pSS risk alleles of rs922483 and rs13277113 indicate an effect of higher expression in males while no effect was observed in females (Additional file 5: Figure S3a, b). For *ARCN1*, the pSS risk allele of rs4938573 was associated with higher expression in females, but not in males (Additional file 5: Figure S3c). Higher expression of *DHX9* was associated with the SLE risk allele of rs12753665 in females, while the effect was opposite in males (Additional file 5: Figure S3d).

In order to corroborate the findings, we performed the interaction analysis for the sex eQTLs and their corresponding genes, but substituting the SNP with one in high (*r* ^2^ > 0.8) or moderate (*r* ^2^ > 0.5) LD, if there were none available with high LD. Six proxy SNPs meeting the criteria were available in the data set. With the exception of rs2077579-*ARCN1* (proxy for rs12753665, *p* = 0.06), all the other SNPs show a significant SNP*sex interaction term and thus confirm the sex-eQTL results (Additional file 3: Table S2).

To investigate if the identified sex-influenced eQTLs are specific to B cells, we analyzed whether the SNPs with significant SNP*sex interaction terms acted as sex eQTLs in other cells or in tissue. For the analysis, we utilized a dataset generated from purified CD14^+^monocytes of 432 genotyped individuals [25] in a similar manner for the analysis in the B cell dataset, as well as the online sex eQTL analysis tool (http://52.37.206.146:8000/) for whole blood based on data from Kukurba et al. [27]. No significant sex-biased regulation of eQTLs by the analyzed SNPs was found (Additional file 6: Table S3), demonstrating cell specificity of the sex-influenced eQTL effects.

The findings of sex-biased eQTLs among susceptibility SNPs for a disease could be a general phenomenon and not connected with a biased sex prevalence of the disease. As a comparison, we therefore analyzed whether there are sex-influenced eQTLs for SNPs associated with autoimmune disease with lesser difference in frequency of affected females/males. For this purpose, we chose type 1 diabetes (T1D), for which the female:male ratio is approximately 1:1 in children [38], and the incidence in adults is 1.5 times higher in men than women (> 15 years of age) [39]. We analyzed the 15 SNPs with a reported genome-wide association with T1D but not concomitantly associated with other diseases with greater differences in female:male ratios [35]. Of these 15, no SNP was associated with a sex-influenced eQTL at the same order of magnitude as the top ones in pSS/SLE, and more importantly, the effect size was lesser for all than for the pSS/SLE-associated SNPs (Additional file 7: Table S4).

In conclusion, we here identify six genes the expression of which is differentially regulated in females compared to males depending on genotype of SLE/pSS-associated SNPs, demonstrating the occurrence of sex-specific eQTL effects of SLE/pSS-associated genetic polymorphisms in defined cells and pinpointing possible genes and pathways which could begin explaining the sex difference in these diseases. We also show that in disease with lower sex bias in prevalence such as T1D, sex eQTLs are less likely to be found among susceptibility SNPs and mediate less effect.

## Discussion

In the present study, we used a directed approach to address the question whether genetic polymorphisms associated with SLE and pSS may contribute to the difference in risk for disease between women and men. We performed an eQTL analysis of SLE and pSS risk loci in primary CD19^+^ B cells, and subsequently narrowed down the question to studying how the context of sex influences eQTL effects of the disease-associated SNPs in B cells. We identified five novel sex-independent eQTLs in B cells with strong effects, which to our knowledge have not been reported previously. In addition, we found sex-influenced eQTL effects for seven of the SLE/pSS SNPs with differential allelic expression effects of six genes between women and men. Four of the sex-dependent effects were exerted by SNPs that also had sex-independent effects on other genes in the same region. By using a cell type specific dataset, we are limiting the spectrum of possible regulatory effects of the disease-associated SNPs and can obtain a more specific signal. Thus, we have been able to identify novel eQTLs in B cells, not reported previously, likely due to lacking power, mixed or other cell types analyzed in preceding studies. The specificity of the observation was also confirmed in that the signals were not present in monocytes or whole blood. By taking only taking the top SLE/pSS-associated SNPs into consideration for the *cis*-eQTL analysis, we were able to minimize the number of tests, and thus the number of type II errors that may arise from multiple comparisons. Interestingly, sex-influenced eQTL effects were substantially less significant and with less effect size for polymorphisms associated with type 1 diabetes, indicating that a high amount of sex-specific eQTL effects of disease-associated polymorphisms is specific for diseases with large differences in the frequency of affected females/males.

Interestingly, the sex-interacting eQTLs detected in this study displayed mostly opposing directions of their effects on expression in men compared to women. While similar observations have been reported previously [28], the underlying mechanisms remain unknown. When considering the pSS/SLE SNPs that showed a significant sex-specific eQTL, most of them are located in non-coding or intergenic regions. These polymorphisms could be responsible for repression or enhancement of gene expression. Sex hormones, through their intracellular receptors, can act both as transcription factors and chromatin modifiers, and the bi-directional sex eQTL effect could be brought about both by direct genetic differences in the receptor binding sites, enhancer elements or their surrounding sequences. One possible scenario is that the gene is regulated by both male and female hormones, and that their binding is affected by the disease-associated SNP. In such a scenario, the disease-associated allele could simply increase binding of e.g. female-specific factors involved in transcription, but decrease the binding of male-specific factors, while the alternative allele has the opposite effect. It is also possible that the disease-associated polymorphisms modulate the recruitment of the regulatory machinery via more distant epigenetic mechanisms, leading to differences in the expression of nearby genes through a pathway that is differentially regulated between the sexes, most probably indirectly orchestrated by sex hormones. Notably, studies have indicated that approximately two thirds of genes differentially expressed between sexes lack androgen or estrogen response elements and were not under direct influence of sex hormones, confirming the complexity of sexually dimorphic gene expression [40].

Whether the genes affected by the identified eQTLs are causal in SLE and/or pSS pathogenesis remains to be investigated. Out of the previously unpublished genes regulated by sex-independent mechanisms, *INPP1*, Inositol polyphosphate-1-phosphatase, is one of the enzymes involved in phosphatidylinositol signaling pathways [41], while *MYO1B,* Myosin Ib, is reported to be involved in morphology and protein transport within multi-vesicular sorting endosomes [42]. Of potential interest is the eQTL affecting expression of *SNRPC*. This eQTL has not been studied before in the context of SLE. The small nuclear ribonucleoprotein polypeptide C (*SNRPC*) gene encodes one of the specific protein components of the U1 small nuclear ribonucleoprotein (snRNP) required for formation of the spliceosome. Autoantibodies to these components are frequently found in autoimmune connective tissue disorders, mainly in SLE [43, 44]. Association signals in the *SNRPC* region have been shown to contribute to the association signal from the *HLA* region in SLE [45].

The most significant eQTL effect difference between females and males was found for rs4637409 (proxy SNP for rs10516487 in the *BANK1* locus) and the expression of *SLC39A8*. In females, presence of the SLE risk allele A was correlated with lower expression of *SLC39A8*, while the effect was opposite in males. The *SLC39A8* (Solute carrier family 39 member 8) gene encodes a manganese and zinc transmembrane transporter localized mainly at the cell membrane, but also at the lysosomal and mitochondrial membranes [46]. *SLC39A8* expression is under transcriptional control of the NFκB pathway. While its role in B cells has not been defined, it has been shown to be induced in other immune cells upon microbial challenge, leading to increased intracellular zinc levels [47]. In lung epithelium, *SLC39A8* was found to be essential for zinc-mediated protection against stress-induced cytotoxicity at the onset of inflammation [48]. Reduced expression of *SLC39A8* in female carriers of the SLE-associated rs10516487 risk allele could potentially lead to enhanced inflammatory stress-induced cell damage and increased exposure of intracellular self-antigens.

The *CD74* gene encodes the class II invariant chain, and is well known for its function as a chaperone, which prevents binding of peptides to the MHC class II molecules in the endoplasmic reticulum (ER), promotes their exit from the ER, directing it into the endocytic compartments, and contributes to peptide editing prior to antigen presentation [49]. CD74 has also been shown to be required for B cell maturation and function [50], and plays an additional role as an accessory signaling molecule on the surface of antigen-presenting cells [51]. In the present study, we found decreased *CD74* expression in B cells from females carrying the risk allele of rs10036748 (proxy SNP for the SLE- and pSS-associated rs7708392 in the *TNIP1* locus), which functionally may relate to an altered regulation of the peptide repertoire presented by MHC II. In males, the same allele was associated with increased *CD74* expression.

Regarding functional consequences of the *PXK* region SNP, one study has reported that it influenced the rate of BCR internalization, and that subjects carrying the risk haplotype had a decreased rate of BCR internalization, a process known to impact B cell survival and cell fate [52]. *CTSB* encodes Cathepsin B, a lysosomal cysteine protease with a poorly studied function, but which potentially may play a role in protein turnover [53]. The protein encoded by *ARCN1* encodes a subunit of the COPI intracellular trafficking system and mutations in this gene cause a developmental craniofacial syndrome [54]. No associations with SLE or any other autoimmune diseases have been reported for *CTSB* and *ARCN1*. The RNA helicase A, encoded by the *DHX9* gene, is a known autoantibody-target in SLE [55]. Interestingly, this gene was first discovered in *Drosophila* as a gene which when mutated caused lethality in male zygotes and was found to play a role in X-chromosome dosage compensation in males [56, 57].

A limitation of the study is the small sample size, although to the best of our knowledge there are no other larger or similarly sized datasets currently available in terms of simultaneous genome-wide genotyping and gene expression data from purified B cells. Further limitations are the lack of information regarding age of the study subjects or other possibly relevant covariates to take into account. However, PCA analysis of ancestry informative markers did not identify any significant vectors, indicating the results are not due to hidden population substructures.

We observed SNPs with both sex-independent eQTL effects for one or several genes, while displaying a sex-biased eQTL effect on another gene. In multifactorial, multigenic diseases such as SLE, a wide range of factors are expected to influence the process leading to overt clinical disease. Genes affected by regulatory SNPs in different contexts, such as different tissue, inflammation and hormonal stimulus are likely influencing the pathogenesis either as a more or less simultaneous cascade at some point, or as single events spanning over a period of time. Theoretically, all those events could be necessary for the eventual development of disease. Thus, it is not surprising to find that one polymorphism or a haplotype could regulate different genes in different contexts, however of equal relevance in the pathogenesis.

## Conclusion

In contrast to previous studies of how sex impacts the pathogenetic mechanisms in SLE, pSS, or other autoimmune diseases, we investigated the aspect of interaction between genetic risk loci and sex as a risk factor in leveraging expression of genes in *cis* in B cells. This approach identified several unknown sex-influenced eQTL effects of the disease-associated genetic polymorphisms, and provides an initial insight into how gene-sex interactions may contribute to the sex-bias in systemic autoimmune diseases. Our main finding is that gene expression will be different if the polymorphism is in the context of a female or male cells, and this may explain why the risk for disease is much higher in females, although the frequency of carriers does not differ between women and men. Our findings will also help designing mechanistic studies intended to pinpoint causal pathways in SLE and pSS pathogenesis to gain functional understanding of why the disease risk differs between men and women.

## Additional files

Additional file 1: Figure S1. “Principal component analysis”. Genotypes for 3736 ancestry informative SNPs were used for a principal component analysis (PCA) by running the smartpca program from Eigensoft using standard settings. Five population outliers were identified. Due to this low number, none of them was removed from the analysis. None of the PCs was significant (*p* = 0.68, 0.75, 0.94, 0.95, 0.95 for the first 5 PCs). (a) scatter plots of first four PCs. (b) scatterplots of first two PCs stratified by genotype and sex for each of six markers with significant sex eQTL effects. The plot for a seventh SNP, rs922483, is not shown as the result was highly similar to rs13277113 (r^2^ = 0.83). (PDF 310 kb)

Additional file 2: Table S1. “Number of probes analyzed per SLE/pSS SNP”. List of SLE/pSS SNPs included in the eQTL analysis, with corresponding numbers of gene expression probes analyzed per SNP. Sheet 2 lists the individual probes and corresponding genes. (XLSX 18 kb)

Additional file 3: Table S2. “Analysis of sex-specific eQTLs using LD SNPs”. SNP*sex Interaction analysis of SNPs in high (r^2^ > 0.8) or moderate (r^2^ > 0.5) LD with the sex-interacting SLE eQTLs. (XLSX 10 kb)

Additional file 4: .Figure S2 “eQTL effects of SLE and/or pSS-associated polymorphisms in B cells”. eQTL effects of SLE and/or pSS-associated SNPs in primary naïve B cells. The *p*-value represents the significance in differential expression between the homozygous group of the non-risk allele and the homozygous group of the risk allele. The rightmost genotype group in each graph denotes the homozygous group of the disease risk allele and is marked by *. (PDF 121 kb)

Additional file 5: Figure S3. “Sex-specific eQTL effects of SLE and/or pSS associated polymorphisms in B cells”. eQTL effects of disease polymorphisms differ between males (left column, blue boxes) and females (right column, red boxes). (a) Genotypic effects of the proxy SNP rs922483 (r^2^ = 1) for the pSS-associated rs2736345 (*BLK/C8orf13* locus) on expression of *CTSB* (ILMN_1696360 probe) in males (*p* = 0.059) and females (*p* = 0.12)*.* (b) Genotypic effects of the SLE-associated rs13277113 (*BLK/C8orf13* locus) on expression of *CTSB* (ILMN_1696360 probe) in males (*p* = 0.16) and females (*p* = 0.19)*.* (c) Genotypic effects of the proxy SNP rs4938573 (r^2^ = 0.865) for the pSS-associated rs7119038 (*CXCR5* locus) on expression of *ARCN1* (ILMN_1699703 probe) in males (*p* = 0.28) and females (*p* = 0.15)*.* (d) Genotypic effects of the proxy SNP rs12753665 (r^2^ = 0.821) for the SLE-associated rs10911363 (*NCF2* locus) on expression of *DHX9* (ILMN_1690965 probe) in males (p = 0.15) and females (*p* = 0.06)*.* The rightmost genotype group in each graph denotes the homozygous group of the disease risk allele and is marked by *. Statistics in boxes represent the Sex * SNP interaction term analysis. (PDF 391 kb)

Additional file 6: Table S3. “Sex-influenced eQTL effects in monocytes and whole blood”. SNPs with SNP * sex interaction terms of FDR < 0.05 analyzed for sex-influenced eQTL effects also in monocytes and whole blood. (DOCX 81 kb)

Additional file 7: Table S4. “Sex-interaction analysis of T1D susceptibility SNPs”. List of SNP*sex interactions (only eQTLs with nominal *p* < 0.05 are shown). Result from a linear regression cis-eQTL analysis with a sex-interaction term, including 15 SNPs associated with T1D and a total of 161 genes included from 1 Mb regions surrounding the SNPs. (DOCX 68 kb)
