# Supplementary figures and images for: Sex influences eQTL effects of SLE and Sjögren’s syndrome-associated genetic polymorphisms
Source: Biol Sex Differ. 2017 Oct 25;8:34. doi: 10.1186/s13293-017-0153-7 (PMC5657123; doi:10.1186/s13293-017-0153-7)

**a**

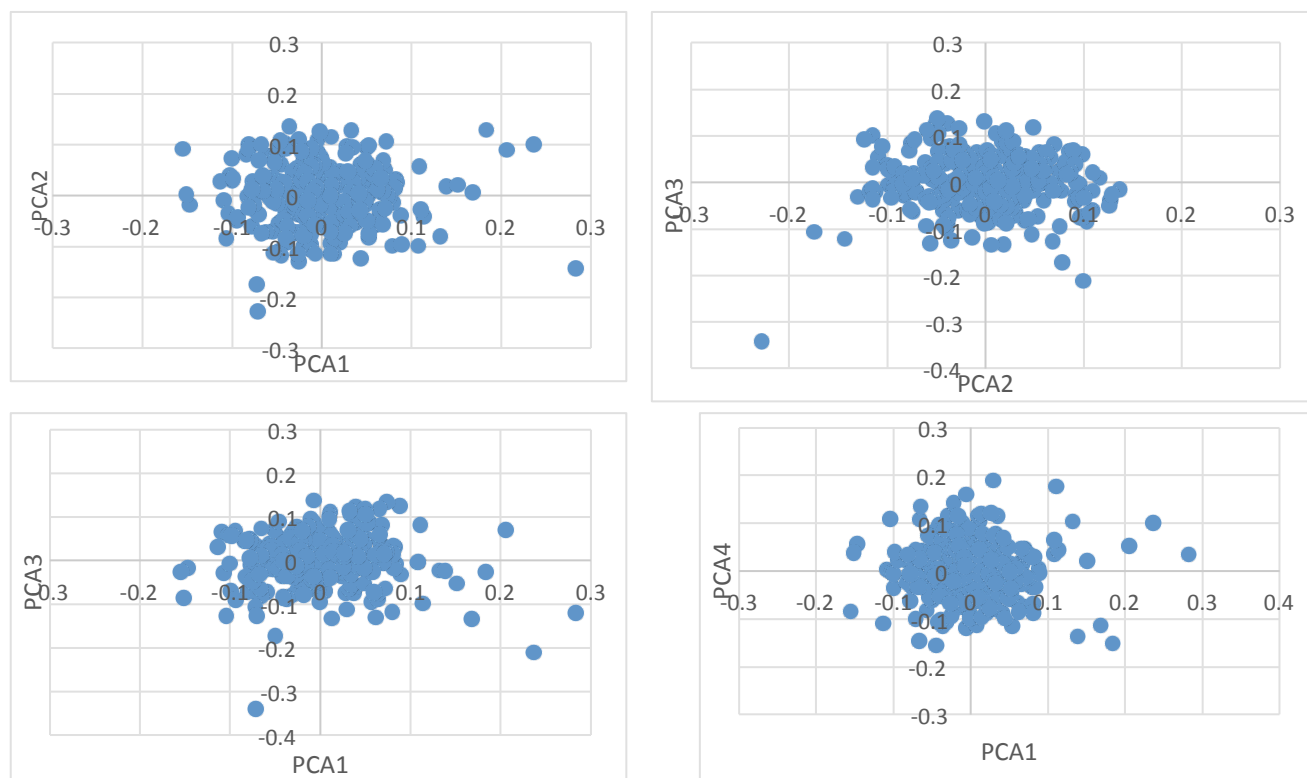

**Figure S1, Lindén *et al* (continues next page)**

**b**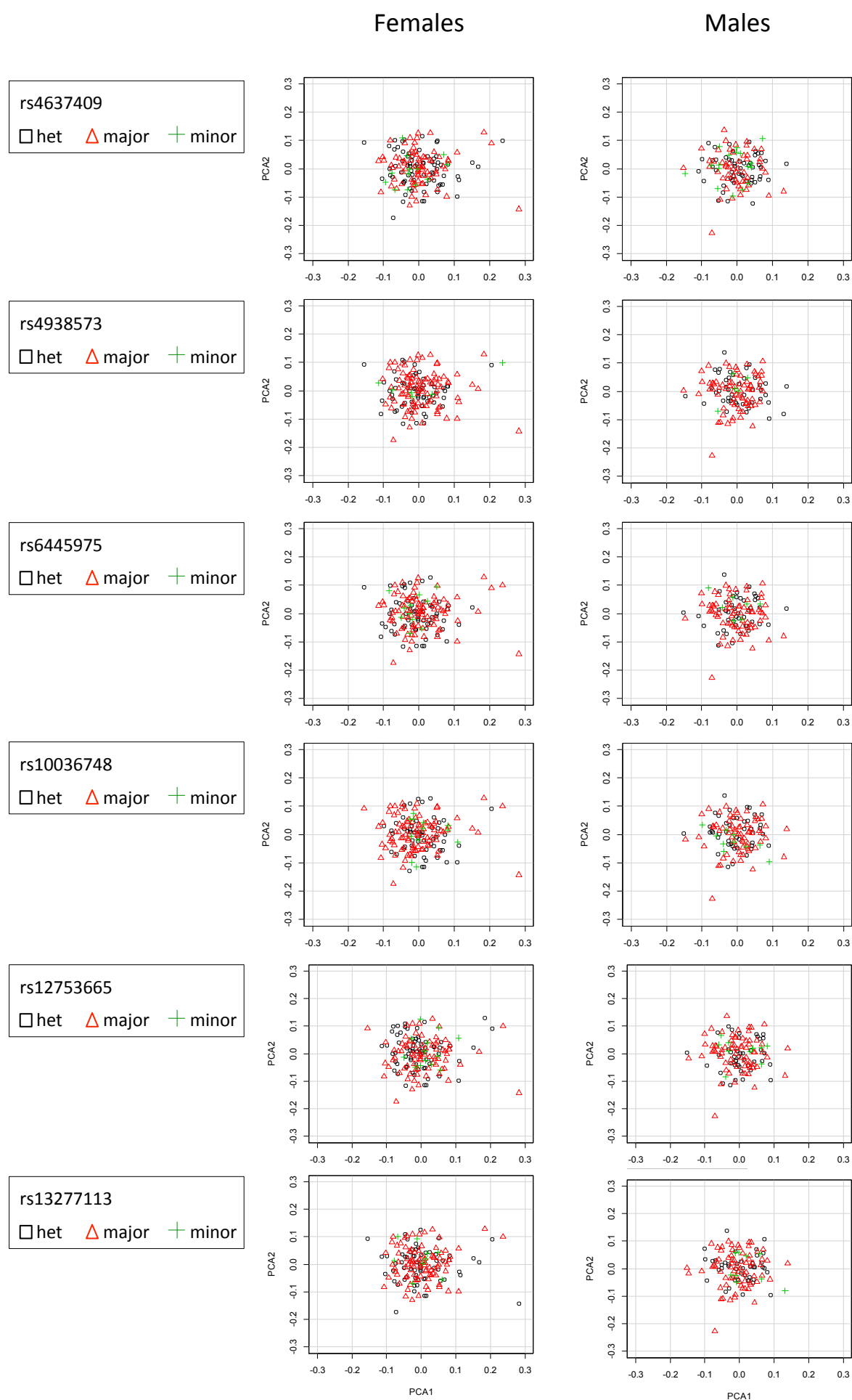**Figure S1, Lindén *et al* (continued)**

Supplement: Supplementary file 1 — “Principal component analysis”. Genotypes for 3736 ancestry informative SNPs were used for a principal component analysis (PCA) by running the smartpca program from Eigensoft using standard settings. Five population outliers were identified. Due to this low number, none of them was removed from the analysis. None of the PCs was significant (p = 0.68, 0.75, 0.94, 0.95, 0.95 for the first 5 PCs). (a) scatter plots of first four PCs. (b) scatterplots of first two PCs stratified by genotype and sex for each of six markers with significant sex eQTL effects. The plot for a seventh SNP, rs922483, is not shown as the result was highly similar to rs13277113 (r2 = 0.83). (PDF 310 kb) [file 13293_2017_153_MOESM1_ESM.pdf]

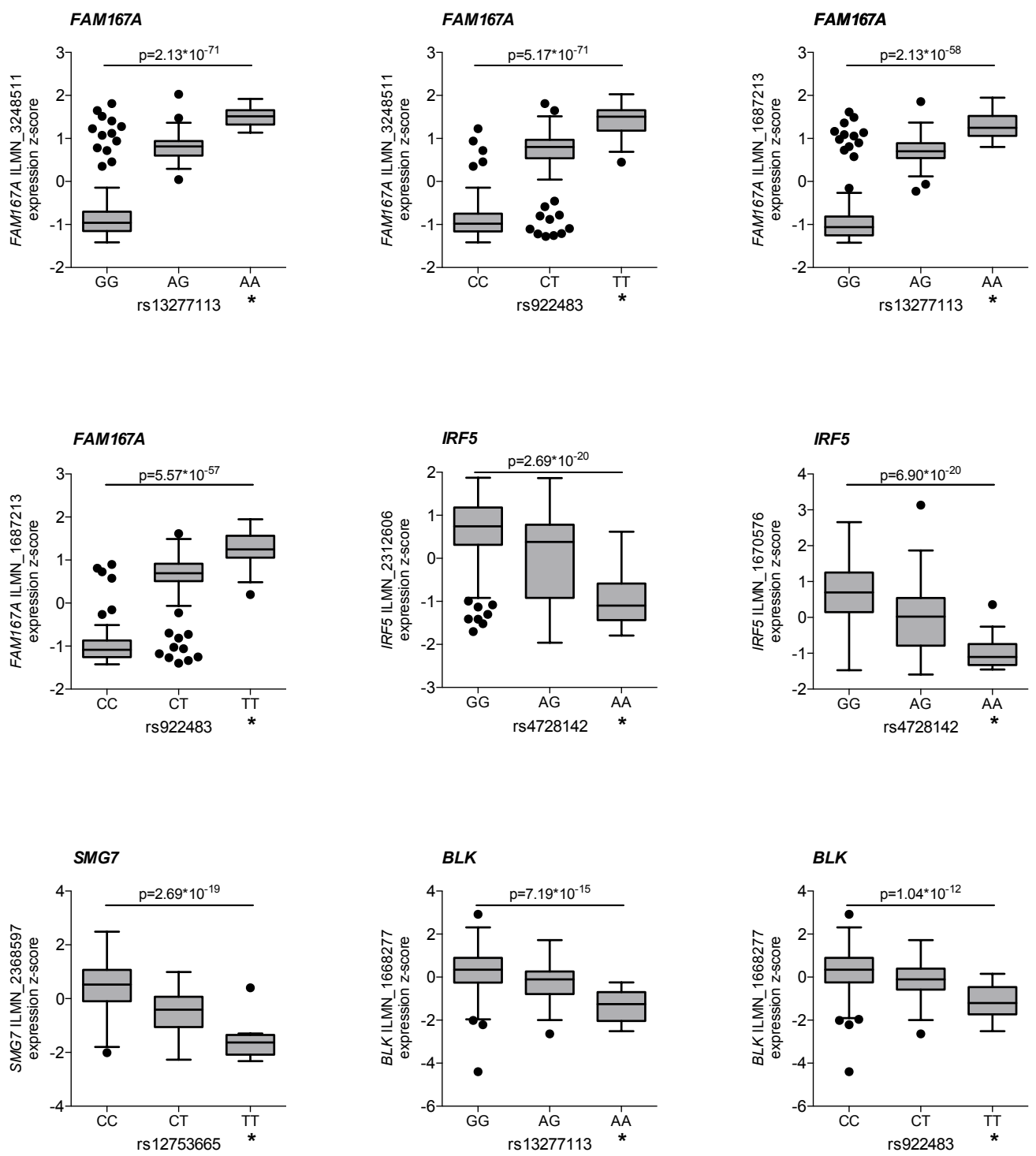

Figure S2, Lindén *et al*

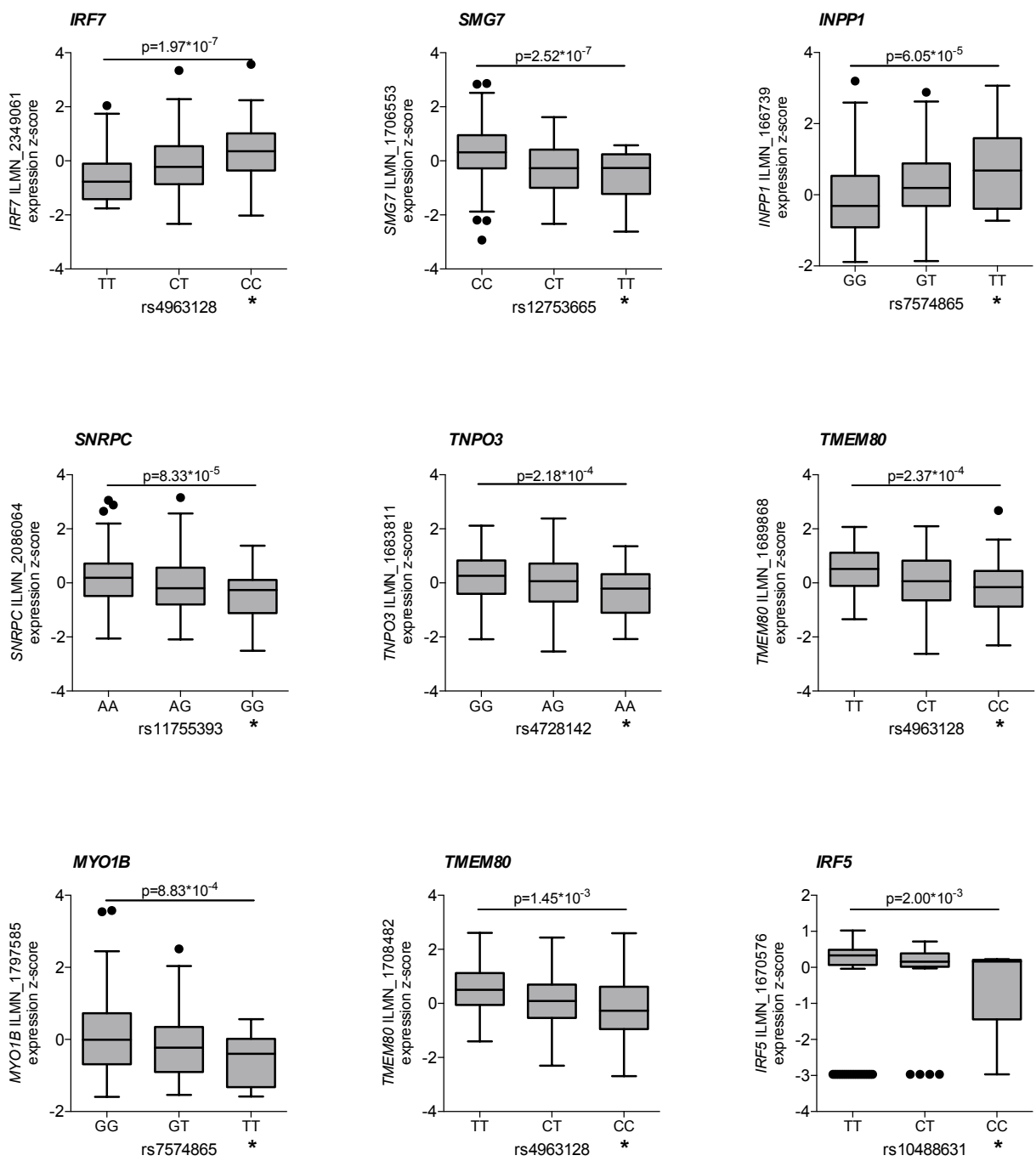

Figure S2, Lindén *et al* (continued)

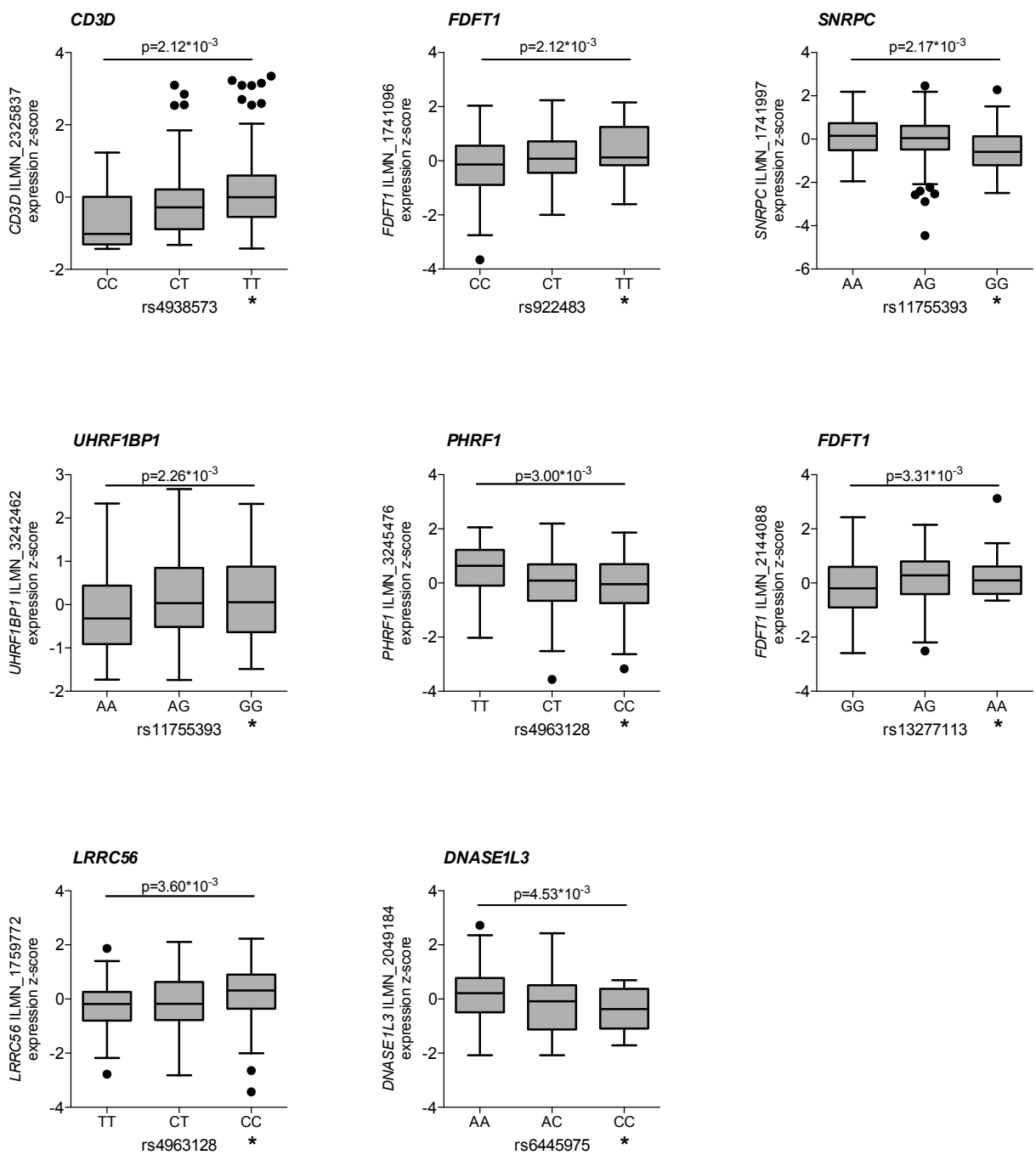

Figure S2, Lindén *et al* (continued)

Supplement: Supplementary file 4 — “eQTL effects of SLE and/or pSS-associated polymorphisms in B cells”. eQTL effects of SLE and/or pSS-associated SNPs in primary naïve B cells. The p-value represents the significance in differential expression between the homozygous group of the non-risk allele and the homozygous group of the risk allele. The rightmost genotype group in each graph denotes the homozygous group of the disease risk allele and is marked by *. (PDF 121 kb) [file 13293_2017_153_MOESM4_ESM.pdf]

**a**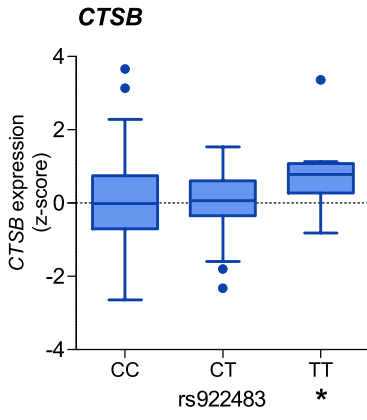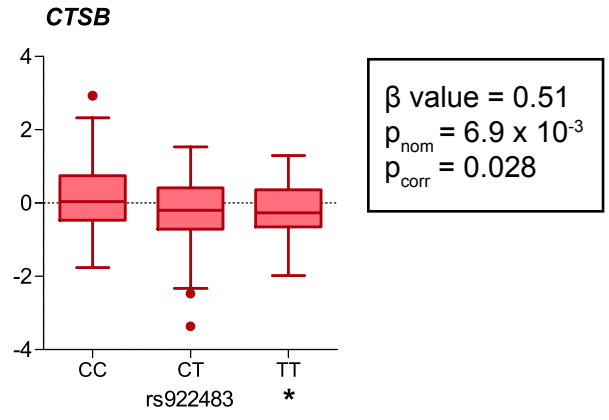**b**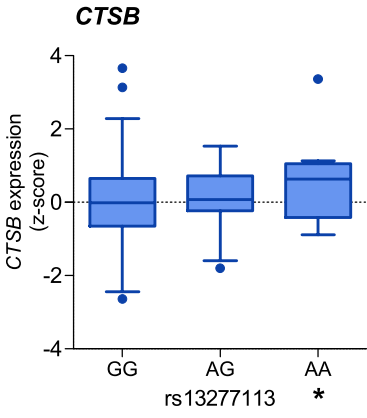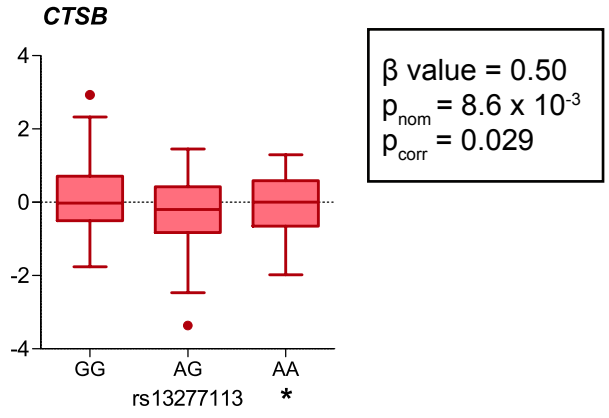**c**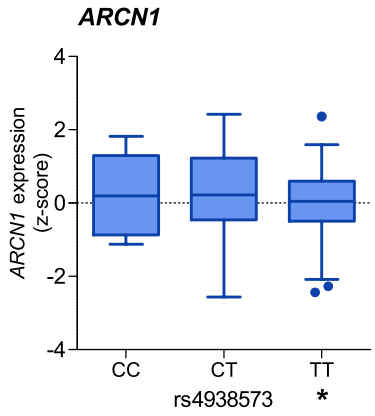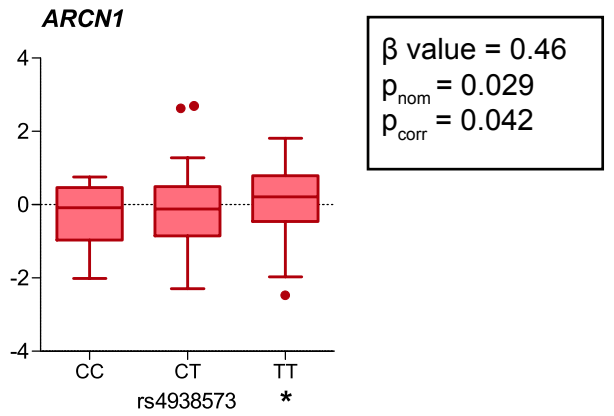**d**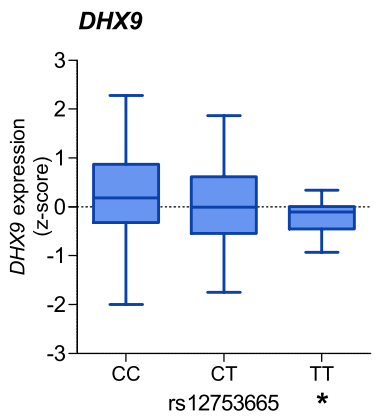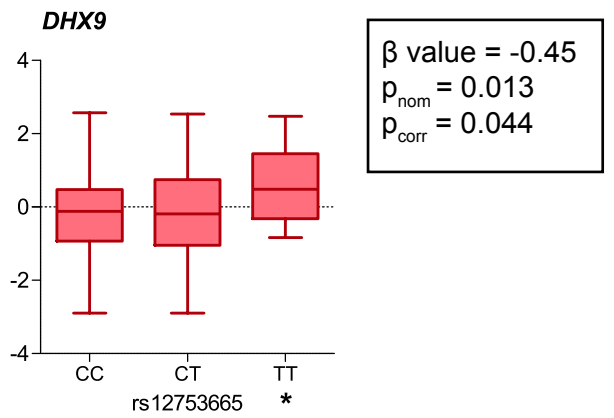**Figure S3, Lindén *et al***

Supplement: Supplementary file 5 — “Sex-specific eQTL effects of SLE and/or pSS associated polymorphisms in B cells”. eQTL effects of disease polymorphisms differ between males (left column, blue boxes) and females (right column, red boxes). (a) Genotypic effects of the proxy SNP rs922483 (r2 = 1) for the pSS-associated rs2736345 (BLK/C8orf13 locus) on expression of CTSB (ILMN_1696360 probe) in males (p = 0.059) and females (p = 0.12). (b) Genotypic effects of the SLE-associated rs13277113 (BLK/C8orf13 locus) on expression of CTSB (ILMN_1696360 probe) in males (p = 0.16) and females (p = 0.19). (c) Genotypic effects of the proxy SNP rs4938573 (r2 = 0.865) for the pSS-associated rs7119038 (CXCR5 locus) on expression of ARCN1 (ILMN_1699703 probe) in males (p = 0.28) and females (p = 0.15). (d) Genotypic effects of the proxy SNP rs12753665 (r2 = 0.821) for the SLE-associated rs10911363 (NCF2 locus) on expression of DHX9 (ILMN_1690965 probe) in males (p = 0.15) and females (p = 0.06). The rightmost genotype group in each graph denotes the homozygous group of the disease risk allele and is marked by *. Statistics in boxes represent the Sex * SNP interaction term analysis. (PDF 391 kb) [file 13293_2017_153_MOESM5_ESM.pdf]
