# Supplementary material for: Sex influences eQTL effects of SLE and Sjögren’s syndrome-associated genetic polymorphisms
Source: Biol Sex Differ. 2017 Oct 25;8:34. doi: 10.1186/s13293-017-0153-7 (PMC5657123; doi:10.1186/s13293-017-0153-7)
Supplement: Supplementary file 6 — “Sex-influenced eQTL effects in monocytes and whole blood”. SNPs with SNP * sex interaction terms of FDR < 0.05 analyzed for sex-influenced eQTL effects also in monocytes and whole blood. (DOCX 81 kb) [file 13293_2017_153_MOESM6_ESM.docx]

| **Chr** | **SNP** | **Reported gene(s) associated with SLE/pSS** | **eQTL Gene** | **Probe ID** | **P**  **SNP** | **P**  **SNP x Sex** | **β value**  **SNP x Sex** | **FDR**  **SNP x Sex** |
| --- | --- | --- | --- | --- | --- | --- | --- | --- |
| **Sorted peripheral monocytes (Fairfax *et al*)** | | | | | | | | |
| 4 | rs4637409 | *BANK1* | *SLC39A8* | ILMN_1695316 | - | - | - | - |
| 5 | rs10036748 | *TNIP1* | *CD74* | ILMN_2379644 | 0.30 | 0.25 | 0.05 | 0.99 |
| 3 | rs6445975 | *PXK* | *PXK* | ILMN_1815063 | 0.26 | 0.04 | -0.06 | 0.74 |
| 8 | rs922483 | *BLK/FAM167A* | *CTSB* | ILMN_1696360 | 0.64 | 0.81 | -0.01 | 0.99 |
| 8 | rs13277113 | *BLK/FAM167A* | *CTSB* | ILMN_1696360 | 0.67 | 0.29 | -0.05 | 0.99 |
| 11 | rs4938573 | *CXCR5* | *ARCN1* | ILMN_1699703 | 0.65 | 0.39 | -0.04 | 0.99 |
| 1 | rs12753665 | *NCF2* | *DHX9* | ILMN_1690965 | 0.42 | 0.35 | 0.02 | 0.99 |
| **Whole blood (Sex-specific eQTL Browser, Kukurba *et al*)** | | | | | | | | |
| 4 | rs4637409 | *BANK1* | *SLC39A8* | - | 0.71 | 0.30 | 0.03 | - |
| 5 | rs10036748 | *TNIP1* | *CD74* | - | 0.55 | 0.77 | -0.008 | - |
| 3 | rs6445975 | *PXK* | *PXK* | - | 1.95e-20 | 0.18 | -0.04 | - |
| 8 | rs922483 | *BLK/FAM167A* | *CTSB* | - | 7.99e-09 | 0.87 | 0.006 | - |
| 8 | rs13277113 | *BLK/FAM167A* | *CTSB* | - | 9.20e-09 | 0.87 | -0.006 | - |
| 11 | rs4938573 | *CXCR5* | *ARCN1* | - | 7.93e-05 | 0.05 | -0.033 | - |
| 1 | rs12753665 | *NCF2* | *DHX9* | - | 0.96 | 0.41 | 0.011 | - |

**Supplementary Table S3.** Analysis of identified sex-specific eQTLs in monocytes and whole blood.
