# Supplementary material for: Sex influences eQTL effects of SLE and Sjögren’s syndrome-associated genetic polymorphisms
Source: Biol Sex Differ. 2017 Oct 25;8:34. doi: 10.1186/s13293-017-0153-7 (PMC5657123; doi:10.1186/s13293-017-0153-7)
Supplement: Supplementary file 7 — “Sex-interaction analysis of T1D susceptibility SNPs”. List of SNP*sex interactions (only eQTLs with nominal p < 0.05 are shown). Result from a linear regression cis-eQTL analysis with a sex-interaction term, including 15 SNPs associated with T1D and a total of 161 genes included from 1 Mb regions surrounding the SNPs. (DOCX 68 kb) [file 13293_2017_153_MOESM7_ESM.docx]

**Supplementary Table S4.** Identification of sex-influenced eQTLs of SNPs associated with type 1 diabetes.

| Chr | SNP*,** | eQTL gene | Probe ID | P nominal | P nominal | β value |
| --- | --- | --- | --- | --- | --- | --- |
|  |  |  |  | SNP | SNP x Sex | SNP x Sex |
|  |  |  |  |  |  |  |
| 20 | rs6043409 | CSNK2A1 | ILMN_2386355 | 0.024 | 0.010 | -0.43 |
| 22 | rs229527 | NCF4 | ILMN_1757361 | 0.0028 | 0.027 | -0.37 |
| 20 | rs6043409 | TBC1 | ILMN_2044572 | 0.034 | 0.030 | -0.37 |
| 14 | rs941576 | EVL | ILMN_1730622 | 0.49 | 0.039 | 0.34 |
| 14 | rs941576 | SETD3 | ILMN_1724504 | 0.020 | 0.040 | 0.34 |
| 20 | rs6043409 | FKBP1A | ILMN_2333367 | 0.0078 | 0.043 | -0.34 |
| 20 | rs6043409 | FAM110A | ILMN_2323944 | 0.060 | 0.045 | -0.34 |

*Of the 15 analysed SNPS, polymorphisms with sex-influenced eQTLs with a nominal p-value <0.05 are included in the table, regardless of FDR.

**rs6427859 and rs7202877 were excluded during quality control due to genotype distribution.
